# Compromised Dynamic Cerebral Autoregulation in Patients With Depression

**DOI:** 10.3389/fpsyt.2019.00373

**Published:** 2019-05-31

**Authors:** Ming-Ya Luo, Zhen-Ni Guo, Yang Qu, Peng Zhang, Zan Wang, Hang Jin, Hong-Yin Ma, Shan Lv, Xin Sun, Yi Yang

**Affiliations:** ^1^Department of Neurology, First Hospital of Jilin University, Chang Chun, China; ^2^Clinical Trial and Research Center for Stroke, Department of Neurology, First Hospital of Jilin University, Chang Chun, China

**Keywords:** depression, dynamic cerebral autoregulation, transcranial Doppler, transfer function, cerebral hemodynamics

## Abstract

**Background:** Patients with depression tend to have various comorbid neurological symptoms, but the mechanisms remain unclear. The purpose of this study was to analyze the characteristics of dynamic cerebral autoregulation in depressed patients.

**Methods:** Patients (aged ≥ 18 years) who were diagnosed with depression [17-item Hamilton Depression Rating Scale (HAMD) > 17] or suspected of depression (HAMD > 7) were enrolled in this study. Medically healthy volunteers were recruited as controls. The subjects also received the 7-item HAMD. We simultaneously recorded noninvasive continuous arterial blood pressure and bilateral middle cerebral artery blood flow velocity from each subject. Cerebral autoregulation was assessed by analyzing the phase difference using transfer function analysis.

**Results:** This study enrolled 54 patients with suspected depression, 45 patients with depression, and 48 healthy volunteers. The mean phase difference values were significantly lower in the patients with depression (F = 9.071, P < 0.001). In the multiple regression analysis, depression was negatively correlated with the phase difference values.

**Conclusions:** Dynamic cerebral autoregulation was compromised in patients with depression and negatively correlated with the depression score. Improving dynamic cerebral autoregulation may be a potential therapeutic method for treating the neurological symptoms of depression.

## Introduction

Depression is the most common psychiatric disorder, a leading cause of disability, and affects nearly 15% of the population (1, 2). Core features of this disorder include depressed mood, loss of interest or pleasure, irritability, change in appetite and sleep, and neurocognitive dysfunctions (3, 4). In addition to suicide ideation and behavior, patients with depression also tend to have comorbid medical illnesses, such as cancer, cardiovascular diseases, and diabetes (5, 6). Depression is associated with an increased risk of stroke morbidity and mortality. These combined conditions generally worsen patient outcomes (7–12).

Despite the prevalence of depression and its considerable burden on global health, knowledge about its pathogenesis remains rudimentary. Previous studies have revealed global and regional changes in the cerebral blood flow of patients with depression compared to healthy individuals (13–15). Cerebral blood flow abnormalities in depression differ in patients, with a varying age of onset (16), disparate responses to antidepressant treatment (17), and diverse family histories (18). Longitudinal research also shows the apparent elevation of regional cerebral perfusion in remissive depression compared to current depression (19). The mechanism of the unusual cerebral blood flow in depressed patients is complex and incompletely understood, and cerebral autoregulation may play a role.

Cerebral autoregulation is the innate ability to maintain appropriate brain perfusion during blood pressure changes. It can be dynamically assessed with transfer function analysis (TFA) between spontaneous fluctuations of arterial blood pressure (ABP) and cerebral blood flow velocity (CBFV) (20, 21). To date, cerebral autoregulation has not been well analyzed in patients with depression. In the present study, we hypothesize that dynamic cerebral autoregulation is compromised in patients with depression, and we use TFA to assess dynamic cerebral autoregulation in depressed patients and explore its relationship with the degree of depression.

## Methods

### Participants and Clinical Assessment

Patients whose first complaint was poor sleep and with 17-item Hamilton Depression Rating Scale (HAMD) scores > 7 were included from the Department of Neurology, First Hospital of Jinlin University, from September 2017 to June 2018. Two blinded clinical psychiatrists evaluated the patients’ mental health status. All patients had never been treated with antidepressants before. Patients with a history of cerebrovascular diseases (that is, cerebrovascular stenosis and stroke), frequent arrhythmias, anemia and unstable blood pressure, and hyperthyroidism were excluded from the study as controls. The patients with hypertension or diabetes took medications, and their blood pressure and blood glucose levels were well controlled. These patients were divided into two groups, those with depression (HAMD ≥ 17) and those suspected of depression (17 > HAMD ≥ 7). Physical health status was assessed using a questionnaire covering cardiovascular, nervous system, thyroid, and metabolic diseases, and information regarding age, smoking, and drinking habits. A total of 48 medically and psychiatrically healthy volunteers were recruited as controls. Liver and kidney function, blood glucose, blood lipid, blood pressure, electrocardiography, transcranial Doppler (EMS-9 PB, Delica, Shenzhen, China), and carotid ultrasound (IU22, Phillips, Andover, MA, USA) tests were used to exclude subjects who did not meet the study standards.

### Cerebral Autoregulation Assessment Monitoring

Before the dynamic cerebral autoregulation examination, all of the patients were instructed to avoid caffeine, nicotine, alcohol, and all kinds of sleep medications for at least 24 h. The assessments were performed in a quiet, dedicated monitoring room with minimal external stimuli. The subjects were instructed to breathe spontaneously and assumed a supine position with a head elevation of 30° when baseline ABP (automatic blood pressure monitor, Omron 711) was measured. Signals were recorded after a 10-min rest. Beat-to-beat ABP was noninvasively recorded through servo-controlled finger plethysmography (Finometer Model 1, FMS, Netherlands), and continuous bilateral middle cerebral artery blood flow velocity was recorded with 2-MHz probes with an insonation depth of 45 to 60 mm attached to a customized head frame (MultiDop X2, DWL, Sipplingen, Germany). Stable end-tidal carbon dioxide (CO2) levels were confirmed through a capnograph with a face mask attached to the nasal cannula. Each participant’s blood pressure and blood flow velocity were recorded for 10 min. The data were then stored for further dynamic cerebral autoregulation examination analysis.

### Analysis of Dynamic Cerebral Autoregulation

The recorded data were analyzed blindly using a laptop computer equipped with MATLAB (MathWorks, Natick, MA, USA). The beat-to-beat alignment of the data was acquired using a cross-correlation function to eliminate possible time lags. By using a cross-correlation function between ABP and CBFV, we may calculate the correlation at each time lag (by sample). We can then find the time lag with the maximum correlation, suggesting that the two signals are synchronized at this time lag. This is considered as the time delay between ABP and CBFV, which is likely caused by the data acquisition devices. We used a third-order Butterworth low-pass filter (cutoff at 0.5 Hz) as an anti-aliasing filter before downsampling the data to 1 Hz. A TFA was applied for evaluating cerebral autoregulation (22). TFA is a frequency domain analysis that calculates the “phase shift” between the CBFV and blood pressure waveforms in the 0.06–0.12 Hz frequency domain to evaluate cerebral autoregulation where the derived parameters were considered most relevant to autoregulation hemodynamics (23). In the current study, a phase shift was accepted for later statistical analysis only if the calculated coherence of one measurement was >0.49 within 0.06–0.12 Hz (24–26), in order to ensure that there was at least 49% linearity between ABP and CBFV. Otherwise, it is invalid to use TFA for the assessment, as it is a linear model.

### Statistical Analysis

The statistical data were analyzed using Statistical Program for Social Sciences version 21.0 (SPSS, IBM, West Grove, PA, USA). Continuous and discrete variables were respectively compared between the patients and healthy controls using analysis of variance. Liner multiple regression was used to explore the association between the phases and characteristics of the patients. The relationships between the phase difference values and the HAMD scores were analyzed using Spearman’s rank-order correlation analysis. Multiple linear regression analysis was used to investigate the effects of the covariates on the phase difference. P values < 0.05 were considered statistically significant.

## Results

### Baseline Characteristics and Phase Difference

The characteristics of the participants are listed in **Table 1**. This study analyzed 45 patients (median age = 47.95 ± 13.30 years, 12 males) with depression, 154 (median age = 47.19 ± 4.48 years, 57 males) suspected of depression, and 48 healthy controls (median age = 47.29 ± 12.24 years, 19 males). The prevalence of hypertension and hyperlipidemia in the patients with depression and those suspected of depression was higher than in the control group.

**Table 1:** Baseline characteristics and phase differences in the patients and controls.

Factors	Depression (n = 154)	Suspected of Depression (n = 45)	Control (n = 48)	F/χ^2^	P
Male, n (%)	57 (37.0%)	12 (26.7%)	19 (39.6%)	2.032	0.362
Age (years)	47.95 ± 13.30	47.27 ± 12.24	47.19 ± 4.48	0.104	0.901
Heart rate	73.10 ± 9.59	75.30 ± 9.79	73.02 ± 8.27	1.007	0.367
Hypertension, n (%)	15 (9.7)	11 (24.4)*	0 (0%)	15.004	0.001
Diabetes, n (%)	7 (4.5%)	2 (4.1%)	0 (0%)	2.254	0.324
Hyperlipidemia, n (%)	12 (7.8%)	5 (11.1%)*	0 (0%)	5.002	0.082
Smoking, n (%)	31 (20.1%)	9 (20.0%)	7 (14.6%)	0.764	0.682
Drinking, n (%)	15 (9.7%)	11 (24.4%)*	0 (0%)	15.004	0.001
Phase difference, degree					
Left hemisphere	48.17 ± 17.15*	42.76 ± 14.01*	56.60 ± 16.00	8.581	<0.001
Right hemisphere	47.78 ± 16.86*	46.38 ± 14.49*	58.25 ± 15.77	8.659	<0.001
Mean ABP, mmHg	89.23 ± 11.84	91.18 ± 9.54	93.26 ± 12.37	2.356	0.097
LCMA velocity	70.05 ± 18.71	66.08 ± 16.39	65.84 ± 14.48	1.583	0.208
RCMA velocity	67.87 ± 16.36	65.72 ± 15.45	64.42 ± 13.10	1.029	0.359
End-tidal CO_2_, mmHg	35.71 ± 3.06	35.78 ± 2.81	34.62 ± 2.94	2.644	0.073

*The difference was statistically significant compared to the control group (P < 0.025).

ABP, arterial blood pressure; LCMA, left cerebral middle artery; RCMA, right cerebral middle artery.

**Figure 1:**
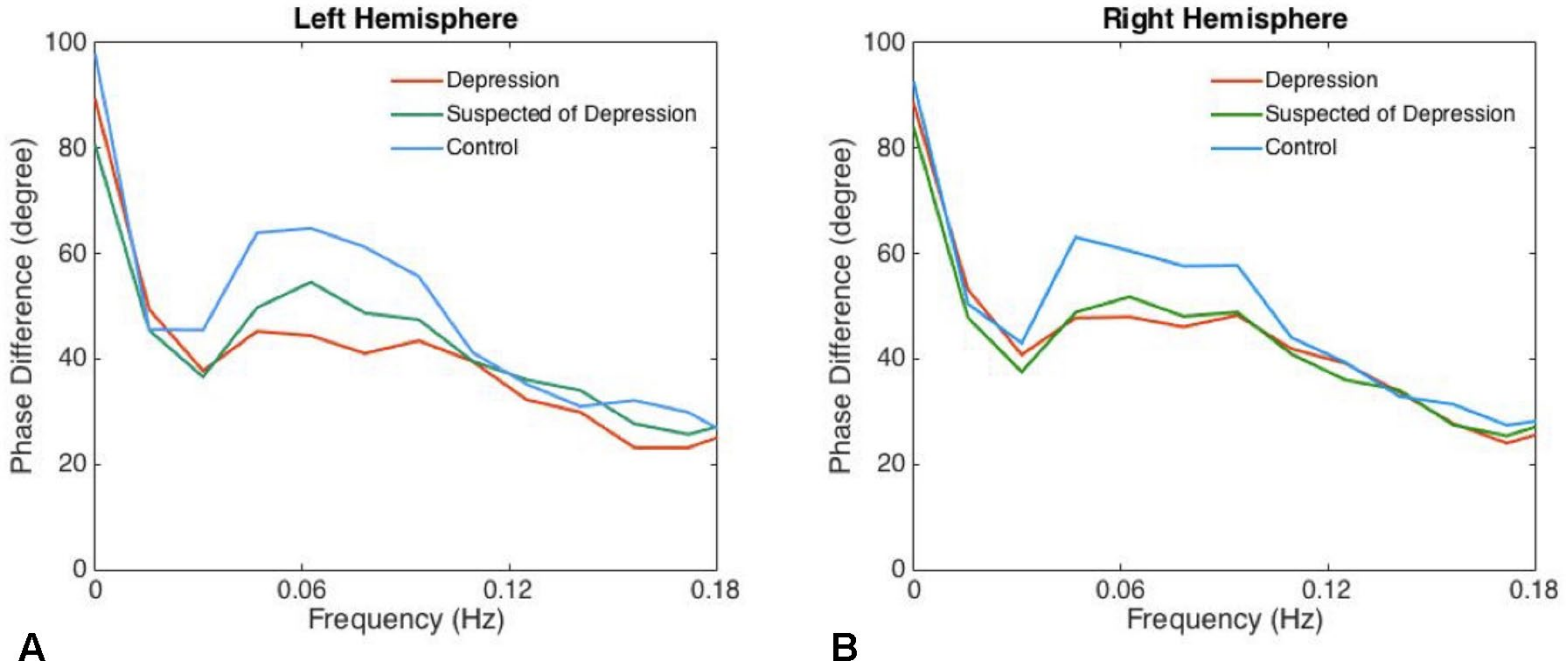
The phase differences of left **(A)** and right **(B)** derived from the transfer function within significant interval 0.06–0.12 Hz are plotted. Phase difference values (parameter of dynamic cerebral autoregulation) were significantly compromised in patients with depression and suspected of depression compared with the controls, indicating an impairment of dynamic cerebral autoregulation in patients with depression.

There was no significant difference between left and right phase difference values in the patients and the controls. The phases in the patients suspected of depression and in those with depression were significantly lower than in the corresponding hemispheres of the healthy controls (**Table 1**, **Figure 1**).

### Multiple Linear Regression Analysis

Age, sex, diabetes, hypertension, hyperlipidemia, tobacco smoking habits, drinking habits, left middle cerebral artery, right middle cerebral artery, mean ABP, and heart rate did not influence the phase averages. However, when the level of depression increased (as evaluated by the HAMD score), the phase difference values were negatively correlated to the HAMD scores (7–17: 95% CI −13.825 to −2.911, P = 0.003; 17: 95% CI −19.725 to −5.802, P < 0.001) (**Table 2**).

**Table 2:** Multiple regression coefficients for the mean phases of the left and right hemispheres.

Factors	Unstandardized coefficients		Standardized coefficients (beta)	95% CI for β		P
	β	Std. error		Lower bound	Upper bound	
Constant	32.594	18.285		–3.433	68.621	0.076
Sex						
Female	Reference					
Male	–1.518	2.384	–0.045	–6.215	3.179	0.525
Hypertension						
No	Reference					
Yes	–3.961	3.786	–0.076	–11.421	3.498	0.209
Diabetes						
No	Reference					
Yes	1.421	5.839	0.017	–10.083	12.926	0.808
Hyperlipidemia						
No	Reference					
Yes	6.383	4.443	0.100	–2.372	15.138	0.152
Smoking						
No	Reference					
Yes	0.250	2.951	0.006	–5.564	6.065	0.932
Drinking						
No	Reference					
Yes	–2.806	3.522	–0.056	–9.746	4.134	0.426
Age	0.022	0.101	0.016	–0.177	0.222	0.826
HAMD						
Control	Reference					
Depression	–8.368	2.770	–0.251	–13.825	–2.911	0.003
Suspected of depression	–12.763	3.533	–0.303	–19.725	–5.802	<0.001
LCMA velocity	–0.100	0.079	–0.109	–0.256	0.056	0.206
RCMA velocity	0.096	0.092	0.093	–0.085	0.278	0.297
Mean ABP, mmHg	0.169	0.094	0.122	–0.017	0.355	0.075
Heart rate	0.123	0.113	0.071	0.100	0.346	0.279
End-tidal CO_2_, mmHg	0.001	0.346	0.001	–0.681	0.682	0.999

ABP, arterial blood pressure; LCMA, left cerebral middle artery; RCMA, right cerebral middle artery.

## Discussion

This study found that dynamic cerebral autoregulation was impaired in patients with depression and the phase difference value was negatively correlated with the HAMD score. Higher levels of depressive symptoms were associated with increased risk of neurological diseases such as stroke or TIA (27). The patients with depression also suffered from dizziness (28). Neurological diseases in patients with depression can be connected to impaired dynamic cerebral autoregulation.

The potential mechanisms underlying this phenomenon are unknown, but there are theoretical possibilities. Recent studies showed that the modulation of neurotransmitters can malfunction in depression (29, 30), and neurotransmitters such as serotonins have a major impact on cerebral vessel tone and could affect cerebral autoregulation (31, 32). In addition, the clinical evidence suggests increased pro-inflammatory markers in patients with depression (increased TNF-α, CRP, and IL-6) (33, 34). These inflammatory factors (35, 36) also can affect the endothelial cells and impair cerebral autoregulation (37). Therefore, depression may influence dynamic cerebral autoregulation through neuroendocrine and immunological/inflammation pathways.

Although various studies (38, 39) indicated that diabetes and hypertension are related to dynamic cerebral autoregulation, we did not find that they have significant influence on cerebral autoregulation. One possible explanation for this disparate finding might be related to the incidence of diabetes and hypertension. This study has a low rate of diabetes and hypertension, and we need to have a larger sample size to further explore their relationship.

In this study, the phase values were correlated with the depression levels. As the HAMD scores increased, the phase difference values (dynamic cerebral autoregulation) tended to decrease. The potential mechanisms are unclear. We supposed that the level of serotonins might be a potential mechanism to explain the relationship between the HAMD scores and the phase values. It has been proven that dysfunction of serotonin is related to major depressive disorder (40). Serotonins also have an impact on cerebrovascular function (41). The study of Edvinsson et al. suggested that serotoninergic projections played a significant role in the regulation of cerebral microvascular tone (42). Nevertheless, a study using PET found that citalopram (a selective serotonin reuptake inhibitor) led to alteration of cerebral hemodynamics (32). In addition, the negative correlation between phase values and the HAMD scale suggests the potential impact of depressive symptoms on dynamic cerebral autoregulation.

The impairment of dynamic cerebral autoregulation in depression indicates that cerebral vascular function may be a therapeutic target of depression. Therefore, improving dynamic cerebral autoregulation may potentially alleviate the neurological symptoms in patients with depression.

This study has some limitations. This was an observational study without in-depth research mechanisms. In addition, the sample size is relatively small. In the future, larger sample sizes and animal studies are needed. In terms of the TFA method, a phase shift is considered valid for further statistical analysis only when the linearity between ABP and CBFV is greater than 49% (24), and TFA is currently the only method that has been studied by multiple centers and standardized by a white paper for the assessment of cerebral autoregulation (23).

## Conclusions

Dynamic cerebral autoregulation was compromised in patients with depression and negatively correlated with depression scores. The mechanism of impaired cerebral autoregulation may play a role not only in the development of cerebrovascular diseases but also as a potential therapeutic method for treating the neurological symptoms of depression.

## Ethics Statement

This study was carried out in accordance with the recommendations of the Declaration of Helsinki and the Ethics Committee of the First Hospital of Jilin University with written informed consent from all subjects. All subjects gave written informed consent in accordance with the Declaration of Helsinki. The protocol was approved by the Ethics Committee of the First Hospital of Jilin University.

## Author Contributions

YY, ML, and ZG drafted the manuscript. ZG and ML revised the manuscript. SL, XS, and HM drew the figures. ZW, PZ, HJ was in charge of acquisition of data. YQ and SL performed the data analysis. ML and PZ performed the statistical analysis. XS and YY conceived and designed the manuscript. All authors read and approved the final manuscript.

## Funding

This article was supported by the National Natural Science Foundation of China (Grant No. 81571123), the National Key R&D Program of China (2016YFC1301600) and JLUSTIRT (2017TD-12) to YY.

## Conflict of Interest Statement

The authors declare that the research was conducted in the absence of any commercial or financial relationships that could be construed as a potential conflict of interest.
